# Measuring students’ self-regulated learning in professional education: bridging the gap between event and aptitude measurements

**DOI:** 10.1007/s11135-015-0255-4

**Published:** 2015-08-26

**Authors:** Maaike D. Endedijk, Mieke Brekelmans, Peter Sleegers, Jan D. Vermunt

**Affiliations:** 1 Faculty of Behavioural, Management and Social Sciences, University of Twente, P.O. Box 217, 7500 AE Enschede, The Netherlands; 2 Department of Education, Utrecht University, P.O. Box 80140, 3508 TC Utrecht, The Netherlands; 3 Faculty of Education, University of Cambridge, 184 Hills Road, Cambridge, CB2 8PQ UK

**Keywords:** Instrument development, Measurement, Self-regulated learning, Professional education, Student teachers, Teacher education

## Abstract

Self-regulated learning has benefits for students’ academic performance in school, but also for expertise development during their professional career. This study examined the validity of an instrument to measure student teachers’ regulation of their learning to teach across multiple and different kinds of learning events in the context of a postgraduate professional teacher education programme. Based on an analysis of the literature, we developed a log with structured questions that could be used as a multiple-event instrument to determine the quality of student teachers’ regulation of learning by combining data from multiple learning experiences. The findings showed that this structured version of the instrument measured student teachers’ regulation of their learning in a valid and reliable way. Furthermore, with the aid of the Structured Learning Report individual differences in student teachers’ regulation of learning could be discerned. Together the findings indicate that a multiple-event instrument can be used to measure regulation of learning in multiple contexts for various learning experiences at the same time, without the necessity of relying on students’ ability to rate themselves across all these different experiences. In this way, this instrument can make an important contribution to bridging the gap between two dominant approaches to measure SRL, the traditional aptitude and event measurement approach.

## Introduction

During the past decades, research on self-regulated learning (SRL) has increased enormously and different models have been developed to conceptualize SRL. Although research has shown that SRL has benefits for academic performance (Cantwell and Moore 1996; Vermunt 2005) and expertise development (Zimmerman 2006), studies also found that students have problems regulating their own learning and that the development from students towards self-regulating professionals does not occur naturally (Evensen et al. 2001). To support students to become self-regulated learners during their professional career, valid instruments are needed to assess the self-regulation strategies students use during their learning (Boekaerts and Cascallar 2006).

Research on SRL conducted so far has mainly focused on students’ SRL in schools for primary and secondary education (Boekaerts and Corno 2005). As a consequence, most of the available instruments to assess student SRL have been developed in traditional school settings to examine the benefits of SRL for academic learning. Far less research has been conducted on how student teachers regulate their learning to teach in postgraduate professional teacher education programmes where two types of learning environments are often combined: a traditional school setting (university) and a professional workplace (practice school) where student teachers do their internship (Endedijk et al. 2012, 2014; Endedijk and Bronkhorst 2014). Moreover, the few available studies into student teachers’ regulation of learning, focused on how student teachers regulate their learning while following a course at the university, rather than how they regulate their learning from practice (e.g., Corrigan and Taylor 2004; Järvenoja and Järvelä 2009). As learning at the workplace is less intentional and planned, does not have pre-set objectives or identifiable outcomes, and is more contextual and collaborative than academic learning (Hodkinson and Hodkinson 2005; Tynjälä 2008), student teachers need to learn different regulation skills to prepare themselves for further professional learning. For example, student teachers need to learn to plan and design their own learning tasks and environment during their internship at the practice school, besides only learning to regulate well-designed and structured learning tasks during their courses at the university (Niemi 2002).

In regulating their own learning to teach, student teachers are thus confronted with multiple and different kinds of learning events as part of their professional training. In order to assess SRL of student teachers, a valid instrument that can deal with the large diversity in learning contexts, but also can discern different qualities of students’ regulation of professional learning is needed (Endedijk et al. 2012). This study makes a contribution to this line of research by examining the validity of an instrument to assess the quality of student teachers’ self regulation strategies across different learning experiences in the context of a postgraduate teacher education programme.

## Theoretical framework

### Defining and measuring self-regulated learning

Following Pintrich, in this study SRL is defined as an “active, constructive process whereby learners set goals for their learning and attempt to monitor, regulate and control their cognition, motivation, and behaviour, guided and constrained by their goals and contextual features in the environment” (Pintrich 2000, p. 453). There is considerable agreement about the importance of SRL, but there has been disagreement about how it can be operationalized and measured in a scientifically useful way (Alexander 2008; Boekaerts and Corno 2005; Zimmerman 2000). As the review of Boekaerts and Corno (2005) showed, the concept of SRL initially has been viewed as a stable individual characteristic resulting in de-contextualised trait-like measurements. In reaction on this static view on SRL as an aptitude, scholars have started to develop new conceptualizations of SRL by using a situated learning approach in which SRL is viewed as a set of dynamic context-dependent activities. Following this situated learning approach, more qualitative and ecologically valid instruments have been developed to measure SRL in real time (Boekaerts and Cascallar 2006; Boekaerts and Corno 2005; Butler 2002; Cascallar et al. 2006; Perry 2002). In current instruments, these two different operationalizations of the concept of SRL can still be recognised. Winne and Perry (2000) made a distinction between instruments that measure SRL as an aptitude and instruments that measure SRL as an event. An event-instrument describes the regulation activities during a specific task. When SRL is measured as an aptitude, a single measurement is used to identify a relative enduring attribute of a person.

Next to a distinction in instruments based on the *operationalization* of SRL, Van Hout Wolters (2000) showed how instruments are divided into on-line and off-line methods. This distinction is related to the *moment* SRL is measured. On-line methods measure SRL during the learning task, off-line methods measure SRL independently from or directly after a learning task. This last distinction is sometimes seen as overlapping with the aptitude-event measurement distinction. Although aptitude instruments are always used off-line, there are also examples of off-line event-measurement. In Table 1, we classified the types of instruments mentioned in several overviews (Boekaerts and Corno 2005; Van Hout Wolters 2000; Van Hout Wolters et al. 2000; Winne and Perry 2000) according to these distinctions.

**Table 1:** Classification of the different types of instruments to measure SRL

	On-line	Off-line
Aptitude		General self-report questionnaires General oral interviews General teacher judgments
Event	Think-aloud methods Eye-movement registration Observation and video-registration of behavior Performance assessment through concrete study tasks, situational manipulations or error detection tasks Trace analysis	Stimulated recall interviews Portfolios and diaries/logs Task-based questionnaire or interview Hypothetical task interview

There has been debate concerning the pros and cons of the different types of instruments mentioned. A review study by Dinsmore et al. (2008) showed, that from the 75 studies, 59 % measured SRL by means of de-contextualised self-reports. This strong reliance on aptitude instruments has often been criticized, because it remains unclear which situations the learners have in mind and which references they have for comparison when completing these questionnaires (Dinsmore et al. 2008; Van Hout Wolters 2000). This may explain why low predicative values of these instruments for learning outcomes and low correlations with on-line methods were found (Veenman 2005). Many authors, therefore, consider the results of self-reports instruments to be poor indicators of the actual regulation activities that students use while studying (Perry 2002; Perry and Winne 2006; Pintrich 2004; Veenman 2005; Winne and Perry 2000). Despite these comments, self-report instruments such as the Motivated Strategies for Learning Questionnaire (MSLQ) (Pintrich and Smith 1993), Inventory of Learning Styles (ILS) (Vermunt 1998), Metacognitive Awareness Inventory (MAI) (Schraw and Dennison 1994), and Learning and Study Strategies Inventory (LASSI) (Weinstein et al. 1987) are still seen as valuable tools for measuring what students perceive to be their general learning preferences, as well as their general motivation and capacity for self-regulation (Perry and Winne 2006; Pintrich 2004; Zimmerman 2008).

An alternative approach is to measure SRL as an event, during an experience or task that is marked by a prior and following event (Winne and Perry 2000). An event-instrument is more suitable for finding relations between specific aspects of real time SRL in authentic contexts (Zimmerman 2008). As Table 1 shows, of the available instruments measuring SRL as an event, some are on-line methods. These on-line methods have the advantage that little information about what happens during the task is lost due to the fact that the measurement actually takes place during the executing of the task (Van Hout Wolters 2006). Despite these benefits, on-line methods are also criticized because of the fact that these instruments influence the learning process of students by for example prompting students to think aloud (Greene and Azevedo 2009). Furthermore, on-line methods only take into account the SRL activities that are performed during the observed learning activity. Moreover, to measure SRL on-line it is essential to have the instrument present during the task. Therefore, for contexts of workplace learning in which students do not learn with the help of pre-set tasks and in which learning is often unplanned (Tynjälä 2008), using on-line instruments for measuring SRL seems to be less relevant and useful.

The off-line event measurement of SRL has less frequently been discussed. Compared to on-line event methods, with these instruments more tacit aspects of SRL can be measured for which the students need some time to recollect what exactly happened during an experience (Howard-Rose and Winne 1993). Of the different types of off-line event instruments, researchers consider portfolios and diaries as one of the most potential and useful instruments to measure SRL in a reliable and valid way (Meeus et al. 2009; Zimmerman 2008). Diaries for example have shown to be equal or even more sensitive than pre- and post-test questionnaires when it comes to measuring changes in SRL in ecologically valid contexts (Zimmerman 2008). From studies in the domain of teacher learning, we know that a digital diary or log is a suitable instrument to collect different kinds of learning experiences (Bakkenes et al. 2010; Hoekstra et al. 2009; Meirink et al. 2009; Van Eekelen et al. 2005; Zwart et al. 2008). On the other hand, researchers also have mentioned that when learners report about unique learning experiences which vary a lot from each other, this also causes standardization problems when the results of individuals need to be compared to each other (Van Hout Wolters 2000).

Concluding, both aptitude and event instruments have the potential to contribute to a deeper understanding of students’ SRL (Howard-Rose and Winne 1993; Winne and Perry 2000). Moreover, no single instrument is capable of capturing all aspects of students’ SRL (Cascallar et al. 2006). The choice of instruments thus depends on the nature of the research problem and the context (Boekaerts and Cascallar 2006; Cascallar et al. 2006; Lonka et al. 2004; Pintrich 2004).

### Criteria for selection of an instrument to measure self-regulation in professional education

Next to the *type of instrument*, a number of different aspects are important to take into account when selecting an appropriate method for assessing SRL (Van Hout Wolters 2000). These include: the *goal* of the assessment, the *type of data* to be collected, the *way of data processing*, the *financial aspects* of the data collection, the *content* of the assessment (which skills are assessed), the *participants* and *context*, the *assessment procedure*, and the *psychometric quality* of the instrument. Below, these aspects will be discussed in the context of professional education in which student teachers’ learning at the educational institute is combined with workplace learning.

The *goal* of the instrument is to diagnose and assess student teachers’ quality of the regulation of their learning across different learning experiences in both the institute and the workplace. Eventually, the instrument should be practical enough to be used on a large scale for repeated measures, to be able to diagnose all student teachers’ quality of regulation during various moments of the teacher education programme. This means that the instrument should generate a *type of data* that is quantitative or easy to quantify, the *processing of the data* should be doable in a short timeframe. Regarding the *financial aspects* the instrument should be able to collect and analyse the data with existing resources of a programme. The *content* of the assessment will be the actual regulation activities that student teachers use when learning to teach. The *participants* are student teachers who learn in different *contexts* of professional education (i.e. institute and workplace). Since the curriculum does not consist of fixed tasks, there is a large variability in student teachers’ learning experiences. This means that the instrument should be able to cover different kinds of learning experiences (e.g., planned and unplanned), in different contexts, with varying duration. This has consequences for the *assessment procedure*: the variation in learning experiences makes it necessary to include multiple learning experiences to give a reliable estimation of student teachers’ quality of regulation. Finally, the *psychometric quality* of the instrument should be high enough to discriminate between different qualities of student teachers’ regulation of learning in a reliable and valid way. In sum, the following criteria can be set for the instrument: it should measure off-line, in a *reliable* and *valid* way, student teachers’ regulation activities during *multiple* and *different kinds of learning events* from the *two dominant contexts* of professional education. Aggregation of these multiple events should make it possible to *discriminate* between different qualities of student teachers’ regulation of learning.

From the different off-line event measurements listed in Table 1, the hypothetical and stimulated-recall interview do not meet these criteria, since they are too labour intensive to use for multiple-event measurements with a relatively large number of participants. Although the portfolio has been suggested as a valid instrument, the use of portfolios varies a lot among student teachers and teacher educators and is therefore in itself not structured enough to collect data of all aspects of the regulation process (Van Tartwijk et al. 2007). As mentioned previously, the diary or log has been used successfully before to collect different kinds of teachers’ learning experiences and was therefore selected as the most suitable instrument for this context.

## Present study

Most empirical studies into SRL have mainly focused on how students regulate their learning in traditional school settings rather than on how students regulate their own learning in a professional educational context where different type of learning environments are combined (traditional school settings and workplace learning). As research on students’ regulation of learning across multiple and different kinds of learning settings is in its infancy (Endedijk et al. 2012; Endedijk and Bronkhorst 2014), an instrument to measure students’ SRL in a professional learning context is needed. The aim of the present study was to develop and assess an instrument to measure student teachers’ regulation of their learning to teach in the context of a postgraduate professional teacher education programme. Based on an analysis of different approaches used to measure SRL and using different criteria, the most suitable instrument for assessing and diagnosing student teachers’ SRL seems to be an off-line multiple event log (diary). Therefore, the main question of our study is: *To what extent can an off*-*line multiple event log be used to measure different qualities of student teachers’ regulation of learning in a valid way?*

The study took place in a 1-year postgraduate professional teacher education programme in the Netherlands. Upon graduation, students receive a subject-matter specific teaching license, that allows teaching in one of the 18 different subjects (e.g., Physics, French language, History) at all levels of secondary education. The programme is similar to postgraduate professional teacher education programmes in other countries (Tryggvason 2009). The programme is a dual programme in which students enroll with a Master’s degree in a specific subject area. Student teachers attend weekly lectures at the university (small group lectures), consisting of general pedagogy classes and subject-specific pedagogy. Next to that, the students are practicing different aspects of teaching at their practice schools or having a paid job as a teacher for half of the study load (workplace learning). Student teachers who have a paid job start from the first day as a teacher at a secondary school. The other student teachers are more gradually exposed to the teaching profession, ranging from observing other teachers and peers, taking over some lessons from an experienced teacher to being responsible for all aspects of teaching.

## Method

We used a two-step procedure to answer the research question. As Cascallar et al. (2006) mentioned, the first step is to have a clear description of the relevant regulation activities that are necessary for students to steer their learning in a specific domain. As this description was not yet available, we needed to develop an open question log aimed at collecting qualitatively rich descriptions of student teachers’ variation in regulation activities across different learning experiences first (pilot study). The qualitative data generated by this instrument have been used to develop, in a second step, a structured question log that is less labour intensive, but still meets criteria of reliability, validity, and discriminative power (main study). The aim of the main study was twofold: First, to replicate the findings found in the pilot study in a new sample with a structured, less labour-intensive version of the instrument. Second, to obtain further indications of reliability, validity and usability of the instrument for teacher education. Below, we will first discuss the method used in the pilot study, followed by the method to test the structured version of the instrument as used in the main study.

### Pilot study

#### Participants

To represent variation in teaching experience, school subject and gender, a random stratified sample strategy was used. Twenty-eight students from the teacher education programme participated. In the final selection, all of the 18 secondary school subjects were included. Nine of the students were male, nineteen were female. The average age of the student teachers was 29 years (SD = 6.1). The student teachers taught on average 7.3 lessons a week at a practice school (SD = 4.0).

#### Instrument

For the measurement of qualitative differences in student teachers’ regulation activities an open question log, called the Learning Report, was developed. In the Learning Report, questions were asked about the three main phases of SRL as described in the conceptual models of Pintrich (2000) and Zimmerman (2000), including forethought, performance, and self-reflection. For the forethought phase questions were inserted about student teachers’ *goal orientation* (Question 2), *sources of self*-*efficacy* (Question 3), and *strategic planning* (Question 4). The questions concerning the performance (monitoring and control) phase described their *learning strategy control* (Question 5) and *monitoring of the learning results* (Question 6). The questions concerning the reflection phase were focused on *self*-*reflection on the learning outcome* (Question 1), *self*-*evaluation of the learning experience* (Question 7) and *inferences for subsequent learning experiences* (Question 8). The questions are listed in Appendix 1. To check whether student teachers would understand the questions of the open question log and whether the instrument was easy in use, three student teachers were asked to fill out the Learning Report (face validity) in a pre-pilot study. Based on their evaluation, some small adaptations were made. To collect information from multiple events, the whole instrument consisted of six Learning Reports in which student teachers could report their six self-chosen learning experiences. The number of six experiences was chosen as this ensured us that we could collect at least two experiences from each context (university and workplace) per participant. In addition, the findings from our pre-pilot study indicated that reporting six learning experiences was not too much of a burden for the students.

#### Procedure

The student teachers were asked to fill out the Learning Report. They received the instruction to select at least two learning experiences from the university, two from their teaching practice and two free of choice. A total of 133 Learning Reports were collected during a period of 6 weeks. Eighteen student teachers completed all six Learning Reports as required, five completed only a part.

#### Analyses

**Fig. 1:**
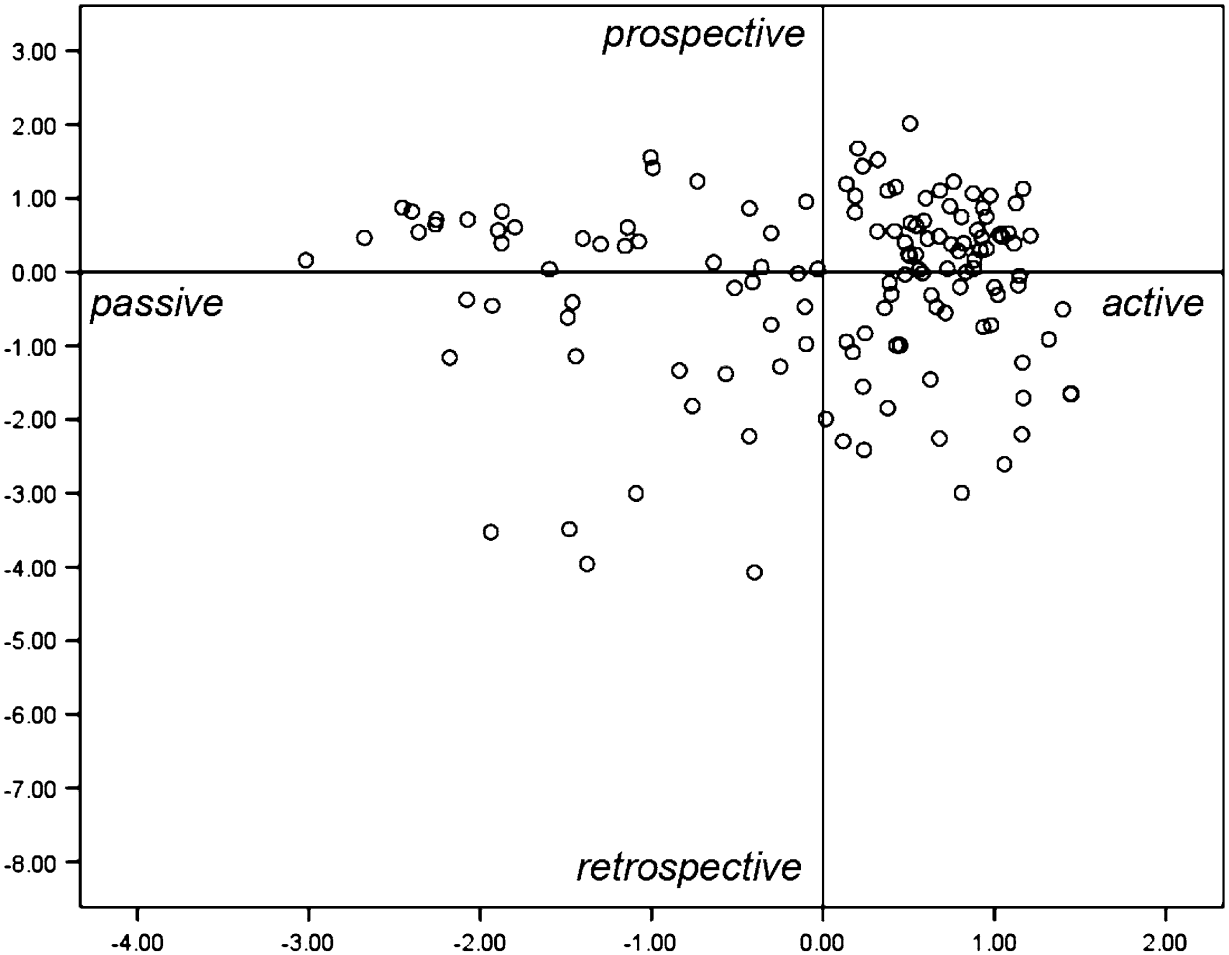
Positioning of all 133 learning experiences of the pilot study on the two dimensions

**Fig. 2:**
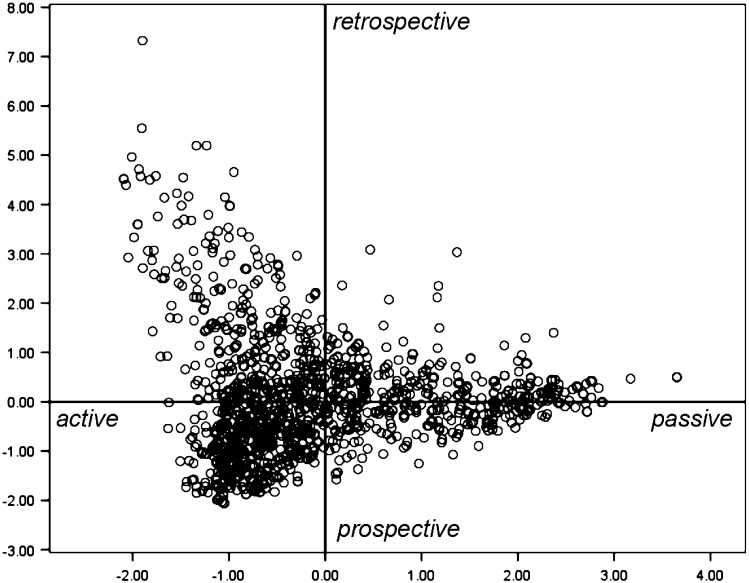
Positioning of all 1294 learning experiences of the main study over the two dimensions

The data of the Learning Reports were analyzed in two phases. In the first phase, fragments of SRL were coded using a set of categories based on the eight questions of the Learning Report. Per question, five to seven categories emerged from the data, representing the qualitative differences in student teachers’ regulation of learning with respect to that particular aspect. The ‘not relevant’ answers were coded as not relevant and these categories were not included in the rest of the analysis (See Appendix 2 for an overview of the categories used for coding and for more detailed information we refer to Endedijk et al. 2012). From the total of 1197 fragments that were coded, 10 % of the fragments (evenly distributed over the variables) was coded by an independent second researcher to compute inter-rater reliability. The Cohen’s κ varied per variable from 0.70 to 1.00, with an overall Cohen’s κ of 0.90. In the second phase, Multiple Correspondence Analysis (MCA, also referred to as homogeneity analysis) on all 133 learning experiences of student teachers was performed (Mair and De Leeuw 2008). MCA is a nonparametric factor analytical procedure, and like parametric factor analysis it orders variables (the categorical scores on the eight regulation questions) along a small number of underlying dimensions. First the number of dimensions was set, using the same procedure as in a regular factor analysis (eigenvalue >1, scree test and meaningful interpretation of dimensions). The outcomes of the MCA show how the different categorical answers of each variable are related to the dimensions. This information was used to interpret the meaning of the dimensions. In the final chart, learning experiences are plotted in the dimensional structure: learning experiences characterized by the same categories are plotted close together, learning experiences with a total different answer patterns are plotted far apart (see Figs. 1, 2). The scores of the objects (in our cases learning experiences) are scaled in such a way, that their variance is equal to their corresponding eigenvalue (Abdi and Valentin 2007).

### Main study

#### Participants

The context of this study was the same post-graduate teacher education programme in the Netherlands as we selected student teachers for our pilot study from. A new complete cohort of student teachers (N = 90) was asked to volunteer in this study. All student teachers were initially willing to participate; however, five student teachers cancelled their participation because of a lack of time, illness, pregnancy, other expectations of the study, and one left the teacher education programme. The total set of participants consisted of 22 male and 63 female student teachers, which covered all of the 18 different secondary school subjects.

#### Instrument

As described earlier, the instrument of the main study, the so-called Structured-Learning Report, was developed on the basis of the results of the pilot study. We used the same eight variables (phrased in eight questions) of the Open Question Learning Report, but this time we developed a more structured approach by using multiple-choice items. The options of the multiple-choice questions reflected the categories from the content analysis of the Open Question Learning Report (see A and B). The number of choices per question varied between five and eight. Besides, for every question there was also the opportunity to use the option “otherwise, namely…”, allowing participants to describe the answer in their own words in case it did not fit the multiple choice options. The Structured Learning Report started with one open question: What did you learn? In this way, the student teachers were able to describe in their own words their reflections on their learning outcomes. Furthermore, the instrument was transformed into a web-based questionnaire, which could be accessed at any moment during the data collection period by the student teacher. Since some of the questions were not relevant for unplanned learning experiences, this online tool made it possible to follow special routes to skip questions in case they were not relevant. Again, students were instructed to complete the Structured Learning Report six times about different experiences.

#### Procedure

The data collection period consisted of three periods of 2 weeks in which student teachers were instructed to report six different learning experiences during every period online in the Structured Learning Report. The periods of data collection took place 3 (T1), 6 (T2), and 9 months (T3) after the start of their programme. After every period of 2 weeks reminders were sent to collect the missing Learning Reports. The student teachers were asked to choose a learning experience that occurred a maximum of 2 weeks earlier; this could be any kind of experience that was part of their development as a teacher. They were stimulated to describe different kinds of learning experiences in the six learning reports: two learning experiences that had taken place (at least partially) in the context of the teacher education institute, two that had taken place (at least partially) in the context of the their work place (practice school), and two of their own choice. Furthermore, they were asked to report planned and spontaneous learning experiences, as well as successful and unsuccessful learning experiences. As a reward they received a personal description of their development as a learner, which they could use for their portfolio that was used in the programme for their final assessment.

In total, 66 student teachers completed all 18 Learning Reports across the three data collection periods, five student teachers participated in just two out of three data collection periods and six student teachers participated only during one data collection period. Four student teachers did not participate during any data collection period. The student teachers differed also in the amount of Structured Learning Reports the filled in per data collection period. Four student teachers completed less than four (out of six) Structured Learning Reports per data collection period, and were therefore excluded from the analyses. This resulted in 75 participants during the first period, 71 participants during the second period, and 68 participants during the last period of data collection. In total 1292 Structured Learning Reports were collected.

#### Analysis

The answers on the first open question (*reflection on the learning object)* of the Structured Learning Report were categorised by the researcher according to the existing coding scheme of the pilot study, which included seven different categories. According to the rule of Cicchetti (1976), for seven categories, 98 observations (2n^2^, in which n is the number of categories) have to be coded by an independent second researcher to gain a reliable interpretation of Cohen’s κ. The inter-rater reliability of the coding of 98 answers to the first question was satisfactory (Cohen’s κ = 0.81). Furthermore, all descriptions of the “otherwise, namely…” from the first data collection period were read and analysed by two researchers. As can be seen in Appendix 2, the frequencies of this category varied between 2.8 and 7.4 % (*M* = 4.6 %) for the different variables. Content analysis of the responses to this question uncovered that student teachers mostly used this option when more than one of the multiple-choice options was applicable to their learning experience or to describe a more specific example of one of the existing options. This indicates that, in general, the list of answer categories resembled the existing variation in regulation activities.

Subsequently, a multiple correspondence analysis as used in the pilot study, was also carried out on the categorical data from of the eight variables to reveal the underlying structure. For this analysis, the category “otherwise, namely…” was treated as a missing value, because the content of what students wrote here varied too much to use it as a separate category. In addition, person-level analysis was carried out to assess the discriminative power of the instrument: individual graphs were made per student teachers per data collection period to display their regulative activities across the six learning experiences. The positions of the set of six learning experiences were categorised based on how the learning experiences were spread over the dimensional structure of the MCA in so-called *regulation configurations*. In this way, also individual differences in student teachers’ regulation of learning were revealed.

## Results

### Pilot study

The answers on the eight open questions were categorised in a total set of 52 categories (5–8 per variable), as are listed in Appendix 1. A detailed overview of the definitions of these categories can be found in Endedijk et al. (2012). The outcome of the multiple correspondence analysis on the data of the pilot study showed that the large variation in student teachers’ regulation activities could be described in terms of an underlying structure of two dimensions. The positioning of all learning experiences on these two dimensions is pictured in Fig. 1.

The first found dimension (horizontal) underlying the data represents (or reflects) a distinction between *passively* versus *actively* regulated learning experiences (Endedijk et al. 2012). Learning experiences that were *passively* regulated by the student teachers were characterised by a lack of argumentation for decisions they had made as well as answers that showed that someone else was in charge of the learning process. Furthermore, many aspects of the regulation process were not described at all. *Actively* regulated learning experiences by student teachers were characterized by the purposeful use of learning strategies as well as the active use of information from others during their learning, and a deeper reflection on the learning outcome. Although in previous research this dimension is often named self-regulation versus external regulation (Kaplan 2008; Vermunt 1998), this definition turned out to be less relevant for the present data. In our data set, external and lack of regulation was found at the same side of the dimension. On the other side of the dimension, examples of regulation by the student teacher were found. Therefore, we interpreted this dimension as active versus passive regulation, in which passive regulation included external as well as lack of regulation.

The second dimension separated prospective and retrospective regulation of learning from each other. In a *prospectively* regulated learning experience, the focus of the regulation activities is on the first phase of the learning process. The learning experience was planned, goals were set and an argumentation for choosing a learning strategy was given. The phase after the learning experience received less attention; the monitoring, reflection, and evaluation were more superficial. The *retrospectively* regulated learning experiences were often unplanned, so no goal-setting or deliberate thinking about learning strategy and self-efficacy had taken place. The regulation focused on the monitoring, evaluation and reflection part of the learning process.

Dimensions 1 and 2 explained respectively 45.1 and 33.9 % of the variance in the data. Based on students’ scores on the eight variables (8 questions), the internal consistency of both dimensions was calculated. Both dimensions showed a satisfactory internal consistency: dimension 1, passive versus active regulation, Cronbach’s α = 0.83; dimension 2, retrospective versus prospective regulation, Cronbach’s α = 0.72.

### Main study

#### The dimensional structure

Figure 2 shows the results of the multiple correspondence analysis on the data of the Structured Learning Report. The findings show that the same dimensional structure was underlying the data as found with the Open Question Learning Report used in the pilot study. The first dimension (horizontal) represents a distinction between *passively* and *actively* regulated learning experiences while the second dimension (vertical) reflects a distinction between *retrospective* versus *prospective* regulated learning activities.

Although the categories from the qualitative analysis (Pilot study) were sometimes positioned slightly differently on the two dimensions than the multiple-choice options, the interpretation of the dimensions remained the same. In general, the data are spread in the same way over the two dimensions. The only difference between the findings from the two studies is that in the main study the variation in scores on the second dimension is smaller on the passive side of the first dimension (Fig. 2), than in the pilot study (Fig. 1). Furthermore, Fig. 2 shows that even more than with the Open Question Learning Report, the second dimension prospective versus retrospective regulation particularly separated learning experiences from each other on the *active* side of dimension 1. Dimensions 1 and 2 explained respectively 43.2 and 34.6 % of the variances in the eight variables. Both dimensions showed comparable Cronbach’s α’s as found in the Pilot Study: 0.81 and 0.73 for the passive-active dimension and the retrospective-prospective dimension respectively.

#### Configurations: person-level analysis

To examine to what extent the Structured Learning Report could assess different qualities of student teachers’ regulation of learning in a valid way (discriminative power), we also conducted a person-level analysis. In this person-level analysis, only cases were included of students who handed in at least four Learning Reports during a data collection period from the maximum of six possible. In total 214 cases could be included: 75 for the first data collection period, 71 for the second and 68 for the third period.

**Fig. 3:**
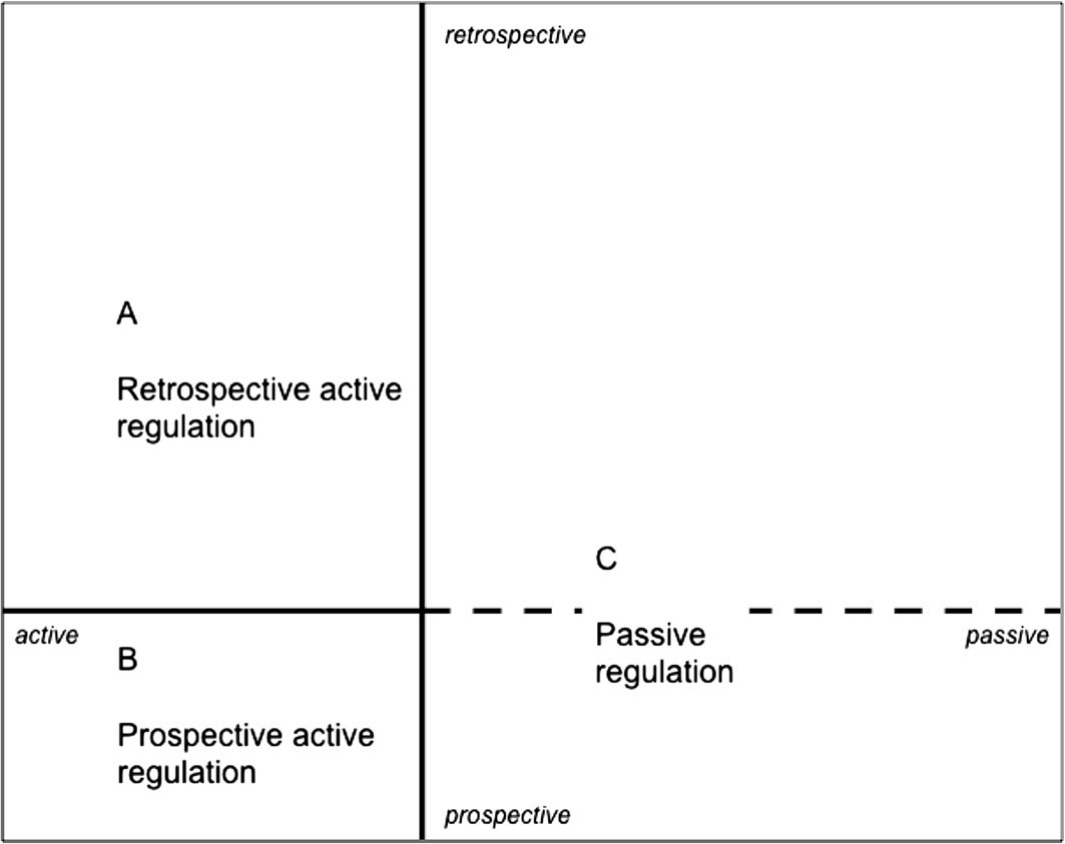
Positioning of the three types of regulation in the dimensional space

To describe the quality of student teachers’ regulation of learning, we had to find a measure that characterizes the quality of regulation of the set of six learning experiences of one measurement moment. The dimensional structure of Fig. 2, shows four quadrants in which all learning experiences are positioned. The position of a learning experience in the quadrant reflects how the learning experience was regulated (active versus passive and prospective versus retrospective). As a first step in this analysis, we classified all single learning experiences into three types of regulation (see Fig. 3):

A. retrospective active regulation (if the score on dimension 1 < 0.0 and dimension 2 > 0.0);
B. prospective active regulation (if dimension 1 < 0.0 and if dimension 2 < 0.0);
C. passive regulation (if dimension 1 > 0.0).

As can be seen in Fig. 3, all learning experiences in the prospective passive quadrant and retrospective passive quadrant are classified as the same type of passive regulation. In total, 27.2 % of the learning experiences were regulation in a retrospective active way, 35.3 % in a prospective active way and 37.5 % were regulated passively.

**Fig. 4:**
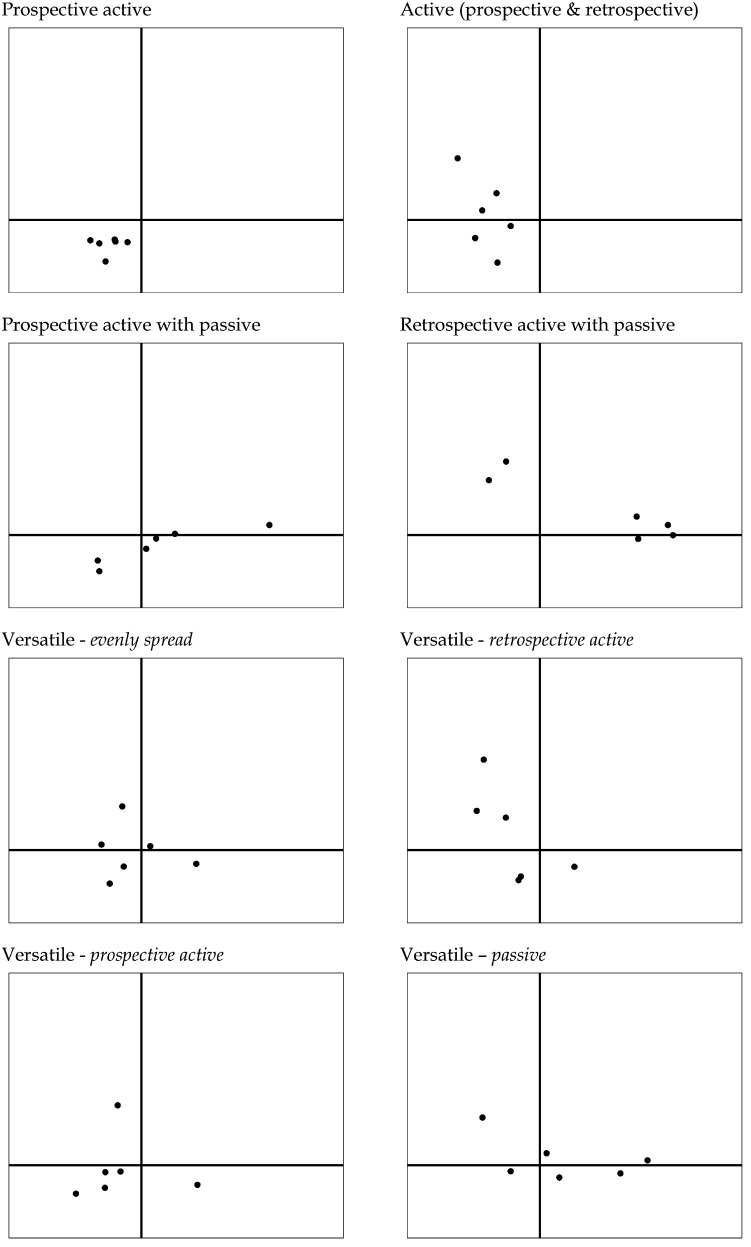
Examples of the eight different regulation configurations. The axes have the same meaning as in Fig. 3: active versus passive and prospective versus retrospective regulation

In the second step, we characterised the combination of the positions of the six learning experiences based on their spreading over these three types of regulation. The student teachers differed in whether their six learning experiences were spread over one, two or three types of regulation. In total eight different typical combinations of how the six learning experiences were spread over the dimensional structure, were identified. These combinations are from now on called configurations (see Table 2). Of every configuration, a typical example is visualized in Fig. 4.

**Table 2:** Frequencies of regulation configurations

Regulation configuration	Frequency	%
Prospective active	2	0.9
Active (prospective and retrospective)	11	5.1
Prospective active with passive	31	14.5
Retrospective active with passive	28	13.1
Versatile	142	66.4
Versatile—evenly spread	27	12.6
Versatile—retrospective active	29	13.6
Versatile—prospective active	41	19.2
Versatile—passive	45	21.0
Total	214	100.0

Almost all student teachers used multiple types of regulation in their learning experiences at one measurement moment: 142 (66, 4 %) cases had their learning experiences spread over all three types of regulation, in 70 (32.7 %) cases the learning experiences were spread over two types of regulation and only in 0.9 % (N = 2) of the cases, just one type of regulation was used. Both cases in this last category, used for all six learning experiences active prospective regulation. This very homogenous regulation configuration was thus named the *active prospective regulation configuration*. Three configurations were based on how the learning experiences were spread over two types of regulation: the *active regulation configuration* (N = 11; 5.1 %), in which active prospective regulation was combined with active retrospective regulation; the *active retrospective with passive regulation configuration* (N = 31; 14.5 %), and the *active prospective with passive regulation configuration* (N = 28, 13.1 %). The cases in which the learning experiences were spread over all three types of regulation were characterised as having a *versatile regulation configuration*. Within this large configuration, a further distinction could be made between the extent to which the six learning experiences were *evenly spread* over the three types of regulation (N = 27, 12.6 %), or whether there was a dominant type of regulation. With respect to the latter type, three different configurations appeared from the findings which we characterised as *active prospectiveregulation* (N = 41, 19.2 %), *active retrospectiveregulation* (N = 29, 13.6 %) or as *passive regulation* (N = 45, 21.0 %).

## Conclusion and discussion

The aim of this study was to develop and assess an instrument to measure student teachers’ regulation of learning to teach across multiple and different kinds of learning events in the context of a postgraduate professional education programme. Based on the literature, we developed a log with structured questions that could be used as a multi-event instrument to determine different qualities of student teachers’ regulation of learning by combining the data from different learning experiences. Furthermore, by combining multiple learning experiences into a regulation configuration we could discriminate different qualities of student teachers’ regulation of learning. The findings from our study clearly show how a classic open event off-line instrument, such as a diary, can be transformed into a structured multiple event learning report that makes it possible to assess the quality of students’ SRL in the context of a postgraduate professional education programme.

The Structured Learning Report was based on an open question version that was developed and examined in a pilot study. This open question version provided rich descriptions of variation in regulation activities, which were used for the formulation of the multiple-choice items of the Structured Learning Report. In case the existing options did not match, it was also possible for student teachers to give their own description. Overall, less than 5 % used this option, which indicates that the options reflected the variation in regulation activities well. Since most of the student teachers that used the alternative option gave an example of one of the existing options, the formulation of some of the multiple choice options could be reconsidered to further improve the instrument. Although in the Structured Learning Report the rich descriptions of the open question version are lost, the reliability of both instruments were satisfactory and comparable.

The underlying dimensional structure that was found with the open version was confirmed with the structured version of the Learning Report. As the open version, the structured instrument measured to what extent students learning experiences were passively or actively regulated, and whether students regulated their learning experiences in a more prospectively or retrospectively way. In the Structured Learning Report, however, the prospective-retrospective dimension mainly separated learning experiences form each other on the active side of the first dimension, resulting in a parabolic curve or horseshoe pattern of the data. This horseshoe pattern is often found in MCA, indicating opposing extreme cases on both sides from general middle group (Greenacre 2006). In Fig. 2, it is clearly visible that the middle group is positioned in the prospective active regulation space with the two ends of the horseshoe high in the retrospective active regulation space and the other in the passive regulation space. To our knowledge, the second dimension (prospective-retrospective) has not been identified before in the literature (see also Endedijk et al. 2012). The existence of this dimension can be explained by the significance of unplanned learning experiences in student teachers’ learning (Hodkinson and Hodkinson 2005). Previous studies into SRL have stressed the crucial role of planning in the active regulation of learning (Eilam and Aharon 2003) and thus emphasising prospective regulation as the classical way of regulating learning. However, the existence of this dimension also shows how unplanned learning experiences can still be regulated in an active way, although more retrospectively.

For the person-level analysis, we first defined three different types of regulation to classify the individual learning experiences: *active retrospective regulation*, *active prospective regulation*, and *passive regulation*. The analysis of the combined set of six learning experiences per person showed that a total of eight regulation configurations were found in the data, reflecting the inter-individual variation in quality of regulation. In almost all of the configurations, multiple types of regulations were combined, indicating the intra-individual differences. With these configurations we showed how the multiple-event instrument could also show differences in quality of regulation on the level of the individual, and in this way be used for feedback purposes in teacher education programmes.

In this study, a multiple event-instrument was developed with the aim of discriminating different qualities of student teachers’ regulation of learning. Results from most single-event instruments describe the quality of regulation during *onespecific* task, restricting the degree to which the results can be generalised to other situations. Existing aptitude instruments, such as self-report questionnaires have been criticized for not measuring students’ SRL in a valid way: it is not clear whether students actually do what they say they did or would do (Veenman 2005). The study of Winne and Jamieson-Noel (2002) showed that students invariably over- and underestimate their use of study tactics. In the Structured Learning Report, the student teachers did not have to rate themselves as a self-regulated learner or to describe how they acted in general over multiple events. The aggregation over multiple experiences was done by deducing regulation configurations based on the results of the multiple correspondence analysis. Another argument against aptitude instruments has been that with these instruments it is not clear which situations the students have in mind while completing the questionnaire (Perry and Winne 2006). The Structured Learning Report strived to overcome these problems by measuring regulation activities in a situated way. Student teachers were asked to select a concrete and recent learning experience, to describe it in their own words and to subsequently pick regulation activities from the multiple choice options that did not match their experience. In conclusion, the multiple-event instrument we developed tried to overcome the regular problems of aptitude instruments without loosing the possibilities for discriminating different qualities of regulation on the level of the individual student teacher.

This choice for a diary type of instrument has also some downsides. As Van Hout Wolters (2000) mentioned, logs and diaries compare students based on different learning experiences. The number of six learning experiences was chosen to catch variations in the regulation of learning within a student teacher, but we did not analyse the role that the number and nature of the selected learning experiences (e.g., contexts and learning tasks) may play in explaining student teachers’ self regulated learning to teach over time. Investigating how the situational variability affect intra- and interpersonal differences in teacher student SRL to teach and how these relations hold over time, using longitudinal designs (Bolger and Laurenceau 2013), would be a fruitful endeavour for future research.

In concluding, the findings clearly show that the instrument we developed is a valid and reliable instrument to diagnose and assess the quality of student teachers’ regulation of learning. As the Structured Learning Report had comparable psychometric qualities, it outperformed the more labour-intensive open question version. The findings of our study thus show how a multiple-event instrument can be used to measure regulation of learning in multiple contexts for various learning experiences at the same time, without the necessity of relying on students’ ability to rate themselves across all these different experiences. In this way, this instrument can make a contribution to bridging the gap between traditional aptitude and event measurement approaches. The procedure that has been used to develop this type of instrument could be exemplary for future research on students’ SRL in professional education programmes other than teacher education, or for other aspects of learning. Findings from these studies can help to validate our findings and contribute to a deeper understanding of students’ SRL in non-traditional school settings.

## Appendix 1: Variables, questions and categories used for coding of the Open Question Learning Report

**Table:**

	Variable	Corresponding question in the instrument	Categories used for coding
1.	Self-reflection on the learning outcome	*What did you learn?*	Rule of thumb; Knowing that; Knowing how; Knowing about myself; Specific teaching practice; Knowing why; Description of an experience
2.	Goal orientation	*Did you plan to learn this, and if so, why did you want to learn this?*	Judgement of current situation; Learning goal; Judgement of current situation and learning goal; Direction of growth with learning goal; Direction of growth with judgement of current situation; No answer
3.	Sources of self-efficacy	*Did you expect to succeed in learning this and what made you think you would (not) succeed in learning this?*	Experience with learning object; Experience with learning strategy; Experience with learning context; Own qualities/efforts; Hope without argumentation; No answer
4.	Strategic planning	*How did you learn this?*	Learning by doing; Reflecting or evaluating; Interacting or getting feedback; Processing information; Applying theory to practice; No answer
5.	Learning strategy control	*Why did you learn it in this way?*	Argument for a way of teaching; Argument for a learning strategy; Part of an instruction; No conscious choice; No answer
6.	Monitoring of the learning results	*How did you realise that you had learned something?*	Reflection on own performance; Experience of what works; Information from (behaviour of) others; Reflection on information of others; Novelty of information; No answer
7.	Self-evaluation of the learning experience	*If you look back, are you completely satisfied, or what would you do differently next time?*	Evaluation of learning strategy; Evaluation of learning context or own behaviour; Evaluation of moment of learning; Completely satisfied; Learning process under control of others; Evaluation of learning content; No answer
8.	Inferences for subsequent learning experiences	*How will you proceed with this learning experience?*	Action plan; Formulating new goal/wish; Consolidation; Improving practice; Applying to practice; No specific changes; No answer

## Appendix 2: Questions and multiple choice items of the Structured Learning Report including frequencies (freq)

1. What did you learn? O*pen question, categorised in terms of the following reflections on the learning content:*

**Table:**

	Multiple choice options	Freq	%
A	Reflection on learning content in terms of a rule of thumb	220	17.0
B	Reflection on learning content in terms of factual knowledge	302	23.4
C	Reflection on learning content in terms of procedural knowledge	78	6.0
D	Reflection on learning content in terms of own learning or identity as a teacher	277	21.4
E	Reflection on learning content in terms of a specific teaching practice	139	10.8
F	Reflection on learning content in terms of theory of practice	194	15.0
G	No reflection in terms of learning, only description of an experience	82	6.3
	Total	1292	100.0

2a. Did you plan to learn this?

**Table:**

	Multiple choice options	Freq	%
A	No, I did not plan to learn this (proceed with question 3).	581	45.0
B	Not specifically for this moment, but I had an intention to learn this.	250	19.3
C	Yes	461	35.7
	Total	1292	100

2b. What was the main reason to learn this?

**Table:**

	Multiple choice options	Freq	%
A	I was unsatisfied about a previous experience	206	15.9
B	I was curious about something	95	7.4
C	Others stimulated me to develop myself in this	125	9.7
D	I wanted to prepare myself for future possible experiences	92	7.1
E	I wanted to practice with something	101	7.8
F	Otherwise, namely…	95	7.4
	*(skipped, because of an unplanned learning experience)*	*578*	*44.7*
	Total	1292	100.0

3. There are different ways to learn things. Not all ways are always applicable to every situation. Please, choose the description that fits your experience best. I learned something by….

**Table:**

	Multiple choice options	Freq	%
A	… I don’t know actually	12	0.9
B	… doing it or experiencing it	352	27.2
C	… experimenting something	174	13.5
D	… evaluating what went well and wrong in my lesson or another situation	93	7.2
E	… analyzing my and others’ role in a situation	85	6.6
F	… getting information	269	20.8
G	… getting feedback from others	186	14.4
H	… observing how others do something	46	3.6
I	Otherwise, namely…	75	5.8
	Total	1292	100.0

4a. Did you choose beforehand this way of learning? *(In the questionnaire, this question is only asked to people who reported a planned learning experience):*

**Table:**

	Multiple choice options	Freq	%
A	No, this was no conscious choice (proceed with question 5)	307	23.8
B	Yes, I thought about that beforehand	403	31.2
	*(skipped, because of an unplanned learning experience)*	*582*	*45.0*
	Total	1292	100.0

4b. You just noticed that you chose your way of learning beforehand. Why did you choose THIS way of learning?

**Table:**

	Multiple choice options	Freq	%
A	I don’t know	19	1.5
B	It is not possible to learn it in another way	125	9.7
C	Someone else suggested to me to learn it this way	74	5.7
D	This was the easiest or the fastest way to learn it	75	5.8
E	Compared with other ways of learning, this way of learning often works well for me	45	3.5
F	Otherwise, namely …	65	5.0
	(skipped, because it was no conscious choice)	307	23.8
	(skipped, because of an unplanned learning experience)	582	45.0
	Total	1292	100.0

5a. Did you expect to succeed in learning this? (In the questionnaire, this question is only asked to people who reported a planned learning experience)

**Table:**

	Multiple choice options	Freq	%
A	Yes	220	17.0
B	No	11	0.9
C	I didn’t know, but I hoped to succeed (proceed with question 6)	178	13.8
D	I didn’t think about that beforehand (proceed with question 6)	55	4.3
	(skipped, because of an unplanned or unintentional learning experience)	828	64.1
	Total	1292	100.0

5b. Why did you expect (not) to succeed in this? *(In the questionnaire, this question is split up in a positive and negative version)*

**Table:**

	Multiple choice options	Freq	%
A	I was (not) confident in myself to succeed	123	9.5
B	I was well prepared	42	3.3
C	The last time I learned something in this WAY, it also worked out well/did not work out well	23	1.8
D	The last time I learned something in this CONTEXT, it also worked out well/did not work out well	5	0.4
E	Otherwise, namely…	38	2.9
	(skipped, because did not think about it)	233	18.1
	(skipped, because of an unplanned or unintentional learning experience)	828	64.1
	Total	1292	100.0

6. At what moment did you realise that you had learned something?

**Table:**

	Multiple choice options	Freq	%
A	I don’t know	20	1.5
B	The moment I experienced that it worked out well	266	20.6
C	The moment I experienced that it did NOT work out well	68	5.3
D	The moment I saw or heard the reaction of others	107	8.3
E	The moment I received feedback	147	11.4
F	The moment I reflected on my experience	165	12.8
G	The moment I realised that I received new information	232	18.0
H	The moment I became aware of my own behaviour	97	7.5
I	Otherwise, namely …	36	2.8
	Missing values (due to a mistake in a skip logic)	154	11.9
	Total	1292	100.0

7a. When you look back on this learning experience, is there something you are unsatisfied about?

**Table:**

	Multiple choice options	Freq	%
A	No (proceed with question 8)	1029	79.6
B	Yes	263	20.4
	Total	1292	100.0

7b. What are you especially unsatisfied about? Retrospectively,….

**Table:**

	Multiple choice options	Freq	%
A	… I would have wanted to learn this earlier in my development	86	6.7
B	… I would have wanted to prepare myself better	31	2.4
C	… I would have wanted to tackle things differently during this experience	49	3.8
D	… I would have liked to learn this in a different way	3	0.2
E	… I would have wanted my students to behave differently	25	1.9
F	… I would have hoped that others would cooperate better	15	1.2
G	Otherwise, namely…	54	4.2
	(skipped, because totally satisfied)	1029	79.6
	Total	1292	100.0

8. How do you proceed with this learning experience?

**Table:**

	Multiple choice options	Freq	%
A	I have no new plans (yet)	121	9.4
B	It did not work out the way I wanted, so I am going to try again	27	2.1
C	I have exactly figured out what I will do next time in a comparable situation	76	5.9
D	I want to consolidate what I have learned	160	12.4
E	I want to improve further what I have learned	308	23.8
F	I want to apply in practice what I have learned	367	28.4
G	I want to try out what I have learned in a different situation	93	7.2
H	Based on what I have learned, I have formulated a new learning goal for myself	85	6.6
I	Otherwise, namely…	55	4.3
	Total	1292	100.0
